# Plant Growth-Promoting Rhizobacteria With ACC Deaminase Activity Enhance Maternal Lateral Root and Seedling Growth in Switchgrass

**DOI:** 10.3389/fpls.2021.800783

**Published:** 2022-01-20

**Authors:** Zhao Chen, Wennan Zhou, Xin Sui, Nan Xu, Tian Zhao, Zhipeng Guo, Junpeng Niu, Quanzhen Wang

**Affiliations:** ^1^Department of Grassland Science, College of Animal Science and Technology, Northwest Agriculture and Forestry University, Xianyang, China; ^2^The State Key Laboratory of Grassland Agro-Ecosystems, College of Pastoral Agriculture Science and Technology, Lanzhou University, Lanzhou, China; ^3^Institute of Animal Husbandry and Veterinary Medicine, Guizhou Academy of Agricultural Sciences, Guiyang, China

**Keywords:** rhizosphere endophytic, *Pseudomonas* sp.Y1, switchgrass cv. blackwell, maternal lateral root, seedling growth

## Abstract

Switchgrass, a C4 plant with high potential as a bioenergy source, is unsteady in yield under sub-optimal conditions. Bacteria containing 1-aminocyclopropane-1-carboxylate synthase (ACC) deaminase can promote plant growth. We isolated bacteria containing ACC deaminase activity from switchgrass rhizosphere using an orthogonal matrix experimental design with four factors (bacterial liquid concentration, bacterial liquid treatment time, nitrogen content, and NaCl) to quantitatively investigate the effects and pairwise interactions on the seedling growth. *Pseudomonas* sp. Y1, isolated from the switchgrass cv. Blackwell rhizomes was selected. We optimized the inoculation bacterial concentration, treatment time, NaCl, and nitrogen concentration for the seedling growth. The optimal bacterial concentration, treatment time, NaCl, and nitrogen content was 0.5–1.25 OD at 600 nm, 3 h, 60–125 mM and 158 mg L^−1^, respectively. *Pseudomonas* sp. Y1 significantly increased the total root length, root surface, shoot length, and fresh and dry weight through an effective proliferation of the number of first-order lateral roots and root tips. This indicated that *Pseudomonas* sp. Y1 has a higher potential to be used as a plant growth-promoting rhizobacteria bacteria.

## Introduction

Plant growth-promoting rhizobacteria (PGPR) has attracted the interest of many researchers because they can promote plant growth and development (Anmol et al., 2021; Kalozoumis et al., 2021). These bacteria promote plant growth through a number of mechanisms including the production of plant growth-promoting substances such as siderophores, various phytohormones, and special activities that include phosphate solubilization and active nitrogen fixing (Gschwendtner et al., 2016; Swamy et al., 2016; Zhang et al., 2016; Wang et al., 2017). These functions can be performed through specific enzymes that induce physiological changes in plants at the molecular level. Among these enzymes, bacterial 1-aminocyclopropane-1-carboxylate (ACC) deaminase plays a vital role in the regulation of ethylene (Valluru et al., 2016). The PGPR with ACC deaminase activity can metabolize ACC into α-ketobutyrate and ammonia; alternatively, ACC will be converted into ethylene (Madhaiyan et al., 2006). Ethylene in the host plant in combination with auxin has a positive effect on plant growth and development, particularly root development (Gschwendtner et al., 2016). Furthermore, the preferred sites of PGPR colonization are the root tips and root elongation zone, where these bacteria can express properties that benefit plants (Nadeem et al., 2012). Soil salinization is a serious stress condition and a land degradation problem in arid and semiarid regions (Sgroy et al., 2009). Salinity is known to increase ethylene production, and increased levels of ethylene aggravate the effect of stress, e.g., by inhibiting root elongation (Pan et al., 2016; Subhan et al., 2020). Bacterial strains containing ACC deaminase activity can partly alleviate the stress-induced ethylene-mediated negative impact on plants. Previous research has shown that inoculation bacteria with ACC deaminase can alleviate the effect of salt stress in various plant species (Barnawal et al., 2013; Nadeem et al., 2013; Subhan et al., 2020; Himadri and Tapan, 2021). Switchgrass (*Panicum virgatum* L.), which is native to North America, is an important perennial rhizomatous C4 species of the tallgrass prairie (Schmer et al., 2011), and has been identified as the primary herbaceous bioenergy crop (McLaughlin and Walsh, 1998; Miesel et al., 2012). Therefore, the discovery of additional endophytic PGPR containing ACC deaminase in switchgrass is significant because these bacteria can promote switchgrass growth under suboptimal conditions.

Nitrogen is an essential macronutrient for plant growth and development, and sufficient nitrogen concentrations can increase plant resistance to stress (Averina et al., 2014). However, indiscriminate use of chemical fertilizers is a significant environmental problem that leads to resource degradation and negatively affects rice paddies and natural wetlands ecological functions (Bodelier et al., 2000). Moreover, there are reports that higher levels of ${\text{NO}}_{3}^{-}$ in the rooting medium stimulate ACC oxidase activity, leading to increased ethylene production (Shaharoona et al., 2006; Wakai et al., 2009). Dipping seeds directly in bacterial liquid is a simple, easy inoculation approach. Kim et al. (2012) reported the optimum concentration of *Burkholderia phytofirmans* strain PsJN in switchgrass was 0.5 at OD 600 nm. The combined effects of nitrogen, NaCl stress, and bacteria containing ACC deaminase on switchgrass is rarely known.

We had isolated the endophytes from switchgrass cv. Blackwell and determined the ACC deaminase activity in the bacteria. Two bacterial species (*Rhizobium* sp. and *Pseudomonas* sp.) were isolated from the switchgrass cv. Blackwell rhizomes (Ma et al., 2015). The objective was to quantitatively investigate the effect of the bacteria on the seedling growth (especially root development) under salt stress and various nitrogen concentrations. We hypothesized that *Pseudomonas* sp.Y1 would significantly increase seedling growth through an increase in the number of first-order lateral roots (NFL) and root tips (NRT). Then, we used an orthogonal matrix design to optimize the inoculation bacterial concentration, treatment time, salinity, and nitrogen concentration for isolated bacteria in the plant seedling growth. To the best of our knowledge, there are limited reports to determine the effect and mechanism of ACC deaminase-producing PGPRs on switchgrass. We aimed to find effective PGPR strains for consideration as biological fertilizers for ameliorating soil salinization incurred in arid regions and to enable the sustainable development of the grass industry.

## Materials and Methods

### Isolation of Bacteria Containing ACC Deaminase

Bacteria were isolated from switchgrass cv. Blackwell grown in the growth chamber in greenhouse of the Grassland Science Department, Northwest A&F Technology University, Shaanxi Province, China. The isolation methods followed Ker et al. (2012), with some modifications. Surface-sterilized (soaked in 70% ethanol for 2 min and 5% NaClO for 15 min) rhizome fragments (2 g each) were macerated in sterile phosphate buffered saline solution. After centrifuging, 1-ml aliquot macerate juice was added to sterile medium containing (per liter) 10 g proteose peptone, 10 g casein hydrolysate, 1.5 g anhydrous MgSO_4_, 1.5 g K_2_HPO_4_, and 10 ml glycerol (PAF medium) and incubated in a rotary shaker at 200 rpm and 28°C for 24 h. Then 1 ml aliquot is removed from the growing culture, transferred to sterile salts minimal DF (Dworkin and Foster, 1958) medium containing 3 mM ACC as the nitrogen source, and incubated in a rotary shaker at 200 rpm and 28°C for 24 h. Four-fold dilutions of this culture were plated onto solid DF salts agar medium containing ACC (500 μmol mL^−1^) and incubated for 48 h at 30°C. Bacterial colonies were chosen based on their colony morphology, further purified, and maintained in the respective tryptic soybean broth (TSB) medium slants at 4°C and in 50% glycerol at −20°C till further use (Ma et al., 2015).

### Identification of the ACC Deaminase-Producing Isolates

The bacterial isolate was genetically identified using 16S rDNA gene sequence analysis. Colony PCR was performed on live cells cultured on solid TSB medium, and the 16S rDNA was amplified using PCR and the bacterial universal primers 27f (5′-AGAGTTTGATCCTGGCTCAG-3′) and 1492r (5′-TACGGCTACCTTGTTACGACTT-3′) primers (Weisburg et al., 1991). The 16S rRNA sequences were determined with an ABI3730-XL DNA sequencer (Sangon Biotechnology Ltd., Shanghai, China). The obtained sequences were compared with the GenBank database using the NCBI Blast program and submitted to GenBank. *Rhizobium* sp. and *Pseudomonas* sp. were isolated. Their 16S rDNA GenBank accession numbers are KM269075 and KJ698416, respectively (Ma et al., 2015). The ACC deaminase activity was assayed according to a modification of the method (Penrose and Glick, 2003), which measures the amount of α-ketobutyrate produced when the enzyme ACC deaminase cleaves ACC. The ACC deaminase production activity of *Pseudomonas* sp. Y1 was 895±35 (nmol α-ketobutyrate per mg protein per h) (Ma et al., 2015).

### Experimental Design

Controlled factors and the levels of the experimental design were assigned using the L16 (4^5^) orthogonal matrix. In the 16 treatments, the four factors tested were the NaCl concentration, bacterial liquid concentration (tested using the OD value at 600 nm), time of bacterial treatment, and nitrogen concentration (Table 1). Prior to adjusting to a moderate OD value, the bacterial ACC deaminase activity was induced according to the method proposed by Donna M. Penrose (Penrose and Glick, 2003; Wang et al., 2017). The ACC deaminase-activated bacteria were dissolved in sterile 0.03 M MgSO_4_ and adjusted to an absorbance of 0.15, 1, and 2 at 600 nm; sterile 0.03 M MgSO_4_ without bacteria was used as the negative control.

**Table 1:** Assignments of the orthogonal matrix L_16_ (4^5^).

**Treatments**	**A (mmol)**	**B (OD value)**	**C (h)**	**D (mg·L^**−1**^)**
1	1 (0)	1 (0)	4 (4.0)	3 (105.00)
2	2 (60)	1	1 (0.5)	1 (13.13)
3	3 (120)	1	3 (2.0)	4 (210.00)
4	4 (180)	1	2 (1.0)	2 (52.50)
5	1	2 (0.15)	3	2
6	2	2	2	4
7	3	2	4	1
8	4	2	1	3
9	1	3 (1.0)	1	4
10	2	3	4	2
11	3	3	2	3
12	4	3	3	1
13	1	4 (2.0)	2	1
14	2	4	3	3
15	3	4	1	2
16	4	4	4	4

*Columns A, B, C, and D present the concentration of NaCI, OD value (concentration of bacteria liquid), treatment time with bacteria liquid and nitrogen content, respectively*.

Switchgrass seeds cv. Blackwell donated by the Institute of Soil and Water Conservation of the Chinese Academy. The seeds were surface-sterilized using 70% ethanol for 1 min, 3% sodium hypochlorite for 10 min, and rinsed 3 times using distilled water. The surface-sterilized seeds were soaked in the respective inoculated for different time periods. After soaking, 15 seeds were placed in each cave (110 mm depth, 180 cc volumes) at a depth of 1 cm. The cave was filled with ~200 g of distilled silica sand that was sterilized at 121°C for 12 h using an electrothermal constant temperature oven (DHG-9140A, Shanghai Yiheng Instrument Co., Ltd., China) prior to use. Each treatment comprised 6 caves, with a total of 90 seeds. The caves were placed in a seedling incubator (LRH-250-GS II, Guangzhou, China) and subjected to an alternating diurnal regime of 16 h of light at 30°C and 8 h of dark at 25°C. The humidity was 50% and illumination intensity was 80%. Seven days after germination, 10 uniform seedlings were selected for further study. The seedlings were irrigated using modified Hoagland's nutrient solution every 4 days; distilled water was added daily for a period of 14 days to compensate for the evaporation loss. Then, the seedlings were irrigated using Hoagland's nutrient solution with different nitrogen contents (1, 1/2, 1/4, and 1/16 times the nitrogen content of modified Hoagland's nutrient solution) until the 3-leaf stage. It takes ~30 days for the seedlings to reach the 3-leaf stage. To study the effect of salt stress on the growth of switchgrass cv. Blackwell seedlings, four NaCl levels (0, 60, 120, 180 mmol L^−1^) were added to the Hoagland's nutrient solution with different nitrogen concentrations, resulting in 16 types of nutrient solutions (Table 1). These nutrient solutions were applied to the seedlings for 2 weeks using the same irrigation method specified above.

### Plant Analyses

After 2 weeks of treatment, 10 seedlings from each treatment were randomly selected for shoot and root length (SL, RL, respectively), measurements; the total sample size was 30 (*N* = 3 replicates × 10 seedlings). The average fresh and dry weights (FW, DW, respectively), of five seedlings (shoot mass) per treatment were determined. There were three replicates for each treatment.

Eight seedlings from each treatment were randomly selected for root analysis. The total RL (TRL), root surface (RS) area, NFL, and NRT were analyzed using the TWAIN PRO (32 bit) root scanner and WinRHIZO root analysis system. The total sample size was 24 (*N* = 3 replicates × 8 seedlings).

### Measurements of Proline, Total Soluble Sugar, and Chlorophyll Contents

Free proline, total soluble sugars, and chlorophyll were extracted from 100 mg of fresh leaves. Proline was estimated by spectrophotometric analysis at 515 nm of the ninhydrin reaction according to Bates et al. (1973). Soluble sugars were analyzed by 0.1 ml of the alcoholic extract reacting with 3 ml freshly prepared anthrone (200 mg anthrone + 100 ml 72% (w:w) H_2_SO_4_) and placed in a boiling water bath for 10 min according to Irigoyen et al. (1992). After cooling, the absorbance at 620 nm was determined in a spectrophotometer. For measuring the chlorophyll content, 100 mg of finely chopped fresh leaves were placed in a capped measuring tube containing 25 mL of 80% acetone and placed inside a refrigerator (4–8°C) for 48 h (Porra, 2002), the chlorophyll content was measured at 645 and 663 nm in a spectrophotometer and calculated using the equation of Porra (2002). There were three replicates for each treatment of the three indices.

### Data Analysis and Statistical Methods

Individual and combined data were analyzed to determine the effects of the four factors on the measured variables (Chatterjee and Hadi, 2006). Simple statistics and the Pearson correlation were calculated, and a multivariate analysis of variance was performed (Gregory, 2014). The NaCl, bacterial liquid, treatment time, and nitrogen were denoted by X_1_ through X_4_, respectively. The dependent variables, i.e., the proline content, soluble sugar (SS), chlorophyll content, RL, shoot length (SL), TRL, RS, FW and DW, NFL, and NRT of the seedlings were denoted by Y_1_ through Y_11_, respectively. These variables were analyzed using pairwise variables (X_1_ and X_2_, X_1_ and X_3_, X_2_ and X_3_) and quadratic and cubic polynomial regression models following the below equation (Chatterjee and Hadi, 2006; Gregory, 2014):


(1) $$\begin{array}{r} Y=\overset{2}{\underset{i=1}{\sum }}\left(\beta_{i\times j+1}X_{i}^{j}\right)+\mu \left(i=1,2;j=1,2\right) \end{array}$$

where β is a constant. Response surface and contour charts are graphed for Y_1_ through Y_11_ with their corresponding X_1_ and X_2_. The coefficients of the models are presented, and all the *P* values were significant at < 0.05 in the models (Supplementary Tables 1–6). For equivalent interactions of X_1_ and X_2_,


(2) $$\begin{array}{r} \frac{\partial Y}{\partial X_{1}}=\frac{\partial Y}{\partial X_{2}} \end{array}$$

Then, quadratic models of X_1_ and X_2_ were obtained, and their curves are presented as ridge lines in **Figure 7**.

The variance ratio contributions (δ) to Y_1_-Y_11_ due to X_1_-X_4_ and their cross products were calculated (Chatterjee and Hadi, 2006).


(3) $$\begin{array}{r} \delta_{ij}=1-\frac{1}{F}\left(\delta =0\text{ when }F≦1\right) \end{array}$$

The statistical analyses and graphical procedures were performed using the Statistical Analysis System (SAS) software (version 8.2, SAS Institute Inc. Cary, North Carolina USA). Differences were considered significant when the mean values of the compared data differed at *p* < 0.05.

## Results

### Isolation, Identification, and ACC Deaminase, IAA Activity of Endophytic Bacteria

#### Variance Ratio Contributions of the Four Factors and Their Cross Products

The FW and DW were significantly and positively correlated with proline, SS and chlorophyll content, and RL and SL (Table 2). The variance analysis results indicated that the four experimental factors significantly influenced the RS), NFL, NRT, TRL, SL, FW, and DW. However, there was no significant time effect on the RS, NRT, or SL, and the effect of nitrogen on the NRT and TRL was not significant. There were significant individual and pairwise effects among the four factors (*p* < 0.001). The bacterial concentration had the greatest effect on the RL, RS, NFL, and NRT (*F*-value = 33.74, 38.71, 391.08, and 5.58, respectively), and the treatment time and nitrogen had the greatest interaction effect on the TRL (*F*-value = 28.61; Tables 3, 4).

**Table 2:** Pearson correlation coefficients of each traits of the seedlings.

	**y_2_**	**y_3_**	**y_4_**	**y_5_**	**y_6_**	**y_7_**	**y_8_**	**y_9_**	**y_10_**	**y_11_**
y_1_	0.112	0.245**	0.086	−0.021	0.103	0.068	0.216**	0.198**	0.108	0.183*
y_2_	1.000	0.118	0.180*	0.166*	−0.051	−0.024	0.434***	0.502***	−0.060	−0.047
y_3_		1.000	0.195*	−0.019	−0.092	−0.072	0.185*	0.195*	−0.111	−0.100
y_4_			1.000	0.227**	−0.062	0.015	0.193*	0.175*	−0.092	−0.080
y_5_				1.000	0.048	0.066	0.271***	0.269***	0.033	−0.021
y_6_					1.000	0.781***	0.023	0.035	0.676***	0.846***
y_7_						1.000	0.023	0.014	0.324***	0.620***
y_8_							1.000	0.982***	−0.002	0.037
y_9_								1.000	0.011	0.039
y_10_									1.000	0.791***

*y_1_, proline content; y_2_, soluble sugar content; y_3_, chlorophyll content; y_4_, root length; y_5_, shoot length; y_6_, total root length; y_7_, root surface; y_8_, fresh weight; y_9_, dry weight; y_10_, number of first-order lateral roots; and y_11_, number of root tips of the seedlings*.

*, **, *** *significant at 0.05, 0.01 and 0.001 probability level, respectively*.

**Table 3:** Variance analyses for the models of shoot length, fresh weight, and dry weight for each experimental factor and among them.

**Source (factors)**		**Shoot lengths**		**Fresh weight**		**Dry weight**	
	**DF**	***F*** **-Value**	***P*****r >** ***F***	***F*** **-Value**	***P*****r >** ***F***	***F*** **-Value**	***P*****r >** ***F***
NaCl	3	3.27	0.0021	44.93	<0.0001	4.77	0.0089
Bacteria	3	38.71	<0.0001	16.53	<0.0001	5.60	0.0042
Time	3	0.57	0.6376	12.76	<0.0001	3.72	0.0238
Nitrogen	3	3.05	0.0326	11.23	<0.0001	3.32	0.0354
NaCl*bacteria	9	2.38	0.0185	21.64	<0.0001	6.58	<0.0001
NaCl*time	9	9.69	<0.0001	40.47	<0.0001	8.34	<0.0001
Bacteria*time	9	3.96	0.0003	Infty	<0.0001	7.49	<0.0001
NaCl*nitrogen	9	25.69	<0.0001	60.15	<0.0001	8.80	<0.0001
Bacteria*nitrogen	9	9.08	<0.0001	Infty	<0.0001	7.88	<0.0001
Time*nitrogen	9	30.27	<0.0001	Infty	<0.0001	10.21	<00.0001
Model	21	6.12	<0.0001	21.48	<0.0001	6.86	<0.0001
*R*-Square		0.7305		0.9455		0.8472	

**Table 4:** Variance analyses for the models of root surface, number of first-order lateral root, root tips, and total root length of the seedlings, for each experimental factor and among them.

**Source (factors)**	**DF**	**Root surface**		**First-Order lateral root**		**Root tips**		**Total root length**	
		***F*** **-Value**	***P*****r >** ***F***	***F*** **-Value**	***P*****r >** ***F***	***F*** **-Value**	***P*****r >** ***F***	***F*** **-Value**	***P*****r >** ***F***
NaCl	3	5.33	0.0020	197.08	<0.0001	3.63	0.0160	24.91	<0.0001
Bacteria	3	33.74	<0.0001	391.08	<0.0001	5.58	0.0015	21.39	<0.0001
Time	3	2.40	0.0729	32.82	<0.0001	0.74	0.5283	8.80	<0.0001
Nitrogen	3	6.67	0.0004	62.54	<0.0001	2.49	0.0655	0.24	0.8688
NaCl*bacteria	9	2.79	0.0063	70.26	<0.0001	2.47	0.0145	7.95	<0.0001
NaCl*time	9	8.83	<0.0001	189.69	<0.0001	3.96	0.0003	12.14	<0.0001
Bacteria*time	9	3.36	0.0014	125.01	<0.0001	3.36	0.0014	13.32	<0.0001
NaCl*nitrogen	9	13.87	<0.0001	179.77	<0.0001	4.67	<0.0001	7.25	<0.0001
Bacteria*nitrogen	9	4.39	<0.0001	115.10	<0.0001	3.06	0.0031	22.71	<0.0001
Time*nitrogen	9	14.84	<0.0001	234.53	<0.0001	3.71	0.0006	28.61	<0.0001
Model	39	11.34	<0.0001	155.75	<0.0001	3.60	<0.0001	271.89	<0.0001
*R*-Square		0.8341		0.9941		0.6146		0.7954	

The four experimental factors (individually and in pairwise combination) significantly influenced the proline and soluble sugar (SS) contents based on the variance analysis. However, the effect of time and the interactions of NaCl with bacteria, and bacteria with nitrogen on chlorophyll were not significant (Table 5). The multivariate analysis of variance showed that NaCl, bacterial liquid, treatment time, and nitrogen significantly affected the dependent variables (Table 6).

**Table 5:** Variance analyses for the models of proline, soluble sugar, and chlorophyll content, for each experimental factor and among them.

**Source**		**Proline**		**Soluble sugar**		**Chlorophyll**	
**(factors)**	**DF**	***F*** **-Value**	***P*****r >** ***F***	***F*** **-Value**	***P*****r >** ***F***	***F*** **-Value**	***P*****r >** ***F***
NaCl	3	185.94	<0.0001	9.01	0.0001	3.67	0.0250
Bacteria	3	9.30	0.0001	3.76	0.0194	6.51	0.0020
Time	3	25.64	<0.0001	3.94	0.0160	0.20	0.3295
Nitrogen	3	10.67	<0.0001	9.13	0.0001	6.26	0.0024
NaCl*bacteria	9	Infty	<0.0001	Infty	<0.0001	1.86	0.1040
NaCl*time	9	Infty	<0.0001	Infty	<0.0001	4.77	0.0008
Bacteria*time	9	Infty	<0.0001	Infty	<0.0001	2.88	0.0167
NaCl*nitrogen	9	Infty	<0.0001	Infty	<0.0001	1.95	0.0888
Bacteria*nitrogen	9	Infty	<0.0001	Infty	<0.0001	1.12	0.3842
Time*nitrogen	9	Infty	<0.0001	Infty	<0.0001	3.01	0.0134
Model	21	57.89	<0.0001	6.46	<0.0001	4.57	0.0002
*R*-Square		0.9520		0.6888		0.7867	

**Table 6:** Multivariate analysis of variance (MANOVA) test criteria and *F* approximations for the hypothesis of no overall effects of the factors.

**Factors**	**Statistic**	**Wilks' Lambda**	**Pillai's trace**	**Hotelling-Lawley trace**	**Roy's greatest root**
NaCl	*F*-Value	56.91	20.01	161.78	458.84
	*P*r > *F*	<0.0001	<0.0001	<0.0001	<0.0001
Bacteria liquid	*F*-Value	17.38	14.16	20.01	32.84
	*P*r > *F*	<0.0001	<0.0001	<0.0001	<0.0001
Time	*F*-Value	18.47	15.97	20.88	39.83
	*P*r > *F*	<0.0001	<0.0001	<0.0001	<0.0001
Nitrogen	*F*-Value	19.24	15.24	23.17	46.26
	*P*r > *F*	<0.0001	<0.0001	<0.0001	<0.0001

*H, Type III SSCP Matrix for NaCl, bacteria liquid, time of treatment, and nitrogen. F Statistic for Roy's Greatest Root is an upper bound*.

The variance ratios show that the bacterial liquid had a maximum contribution with a total value 20.483, followed by NaCl (16.593) (Supplementary Table 7). Nitrogen contributed the least, with a total value of 2.033. The contributions of the pairwise factors for time × nitrogen and bacteria × nitrogen had maximum total values of 13.563 and 13.550, respectively. The pairwise effects in decreasing order were time × nitrogen > bacteria × nitrogen > NaCl × time > NaCl × nitrogen > NaCl × bacteria > bacteria × time. The maximum variance ratio contribution was observed for proline and the minimum for RL, with total values of 15.067 and 8.132, respectively. The bacterial liquid was the highest contributor to the SL (2.705), TRL (2.717), RS (2.693), NFL (2.516), and NRT (2.356). The bacteria × nitrogen contributed largely to proline (2.806), SS (2.169), and SL (2.035). The NaCl × bacteria contribution to the FW was 2.095 (Supplementary Table 7).

#### Pseudomonas sp. Y1 Promotes the Growth of Switchgrass cv. Blackwell Seedlings

**Figure 1:**
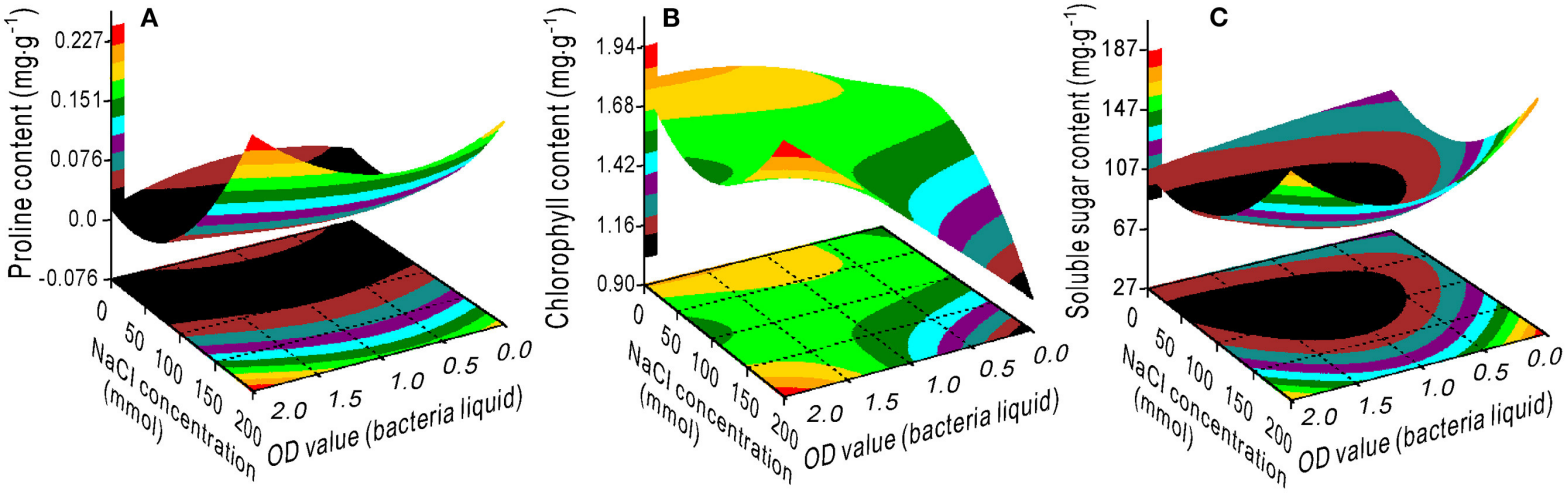
Response surface plots showing the interaction effects on the proline **(A)**, chlorophyll **(B)**, and soluble sugar **(C)** contents in the switchgrass by the NaCl concentration and bacterial liquid.

The response surface plots showed the interaction effects on proline (Figure 1A), chlorophyll (Figure 1B), and SS (Figure 1C) contents from the bivariate regression model analyses. The proline and SS accumulated with an increase in the NaCl concentration. The effect of the bacterial concentration on proline was smaller than the effect of NaCl, which is consistent with the variance ratio contributions; the contributions of NaCl and bacteria to proline were 2.951 and 0.143, respectively (Figure 1A). There was a minimum accumulation value of the SS at 75 mM NaCl and 1.25 OD bacterial liquid concentrations (Figure 1C). The higher the OD value of the bacterial liquid when the NaCl concentration was 200 mM, or the lower the NaCl concentration, the greater the chlorophyll accumulated (Figure 1B).

**Figure 2:**
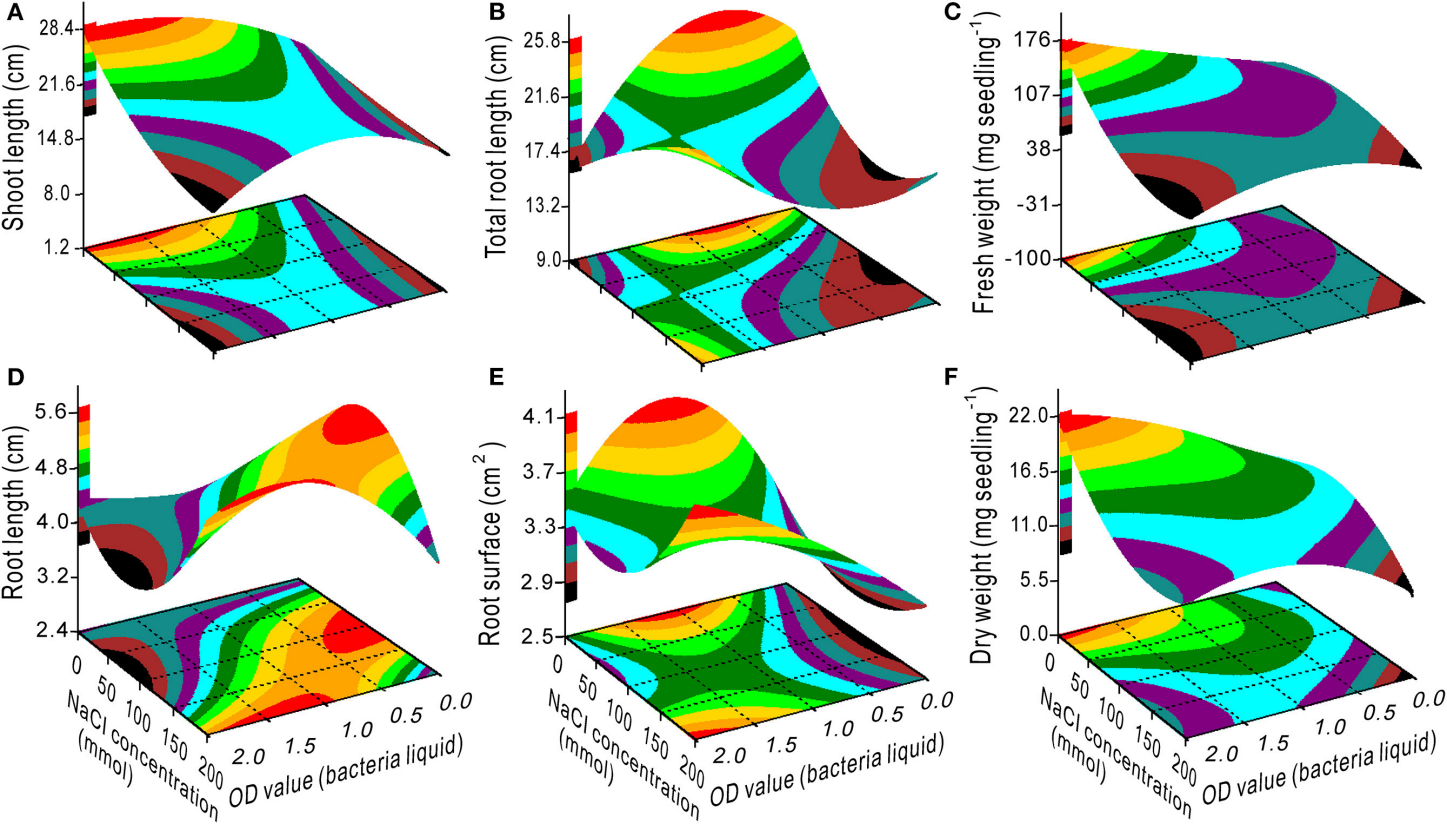
Response surface plots showing the interaction between the NaCl concentration and bacterial liquid on shoot length **(A)**, total root length **(B)**, fresh weight **(C)**, root length **(D)**, root surface **(E)**, and dry weight **(F)**.

**Figure 3:**
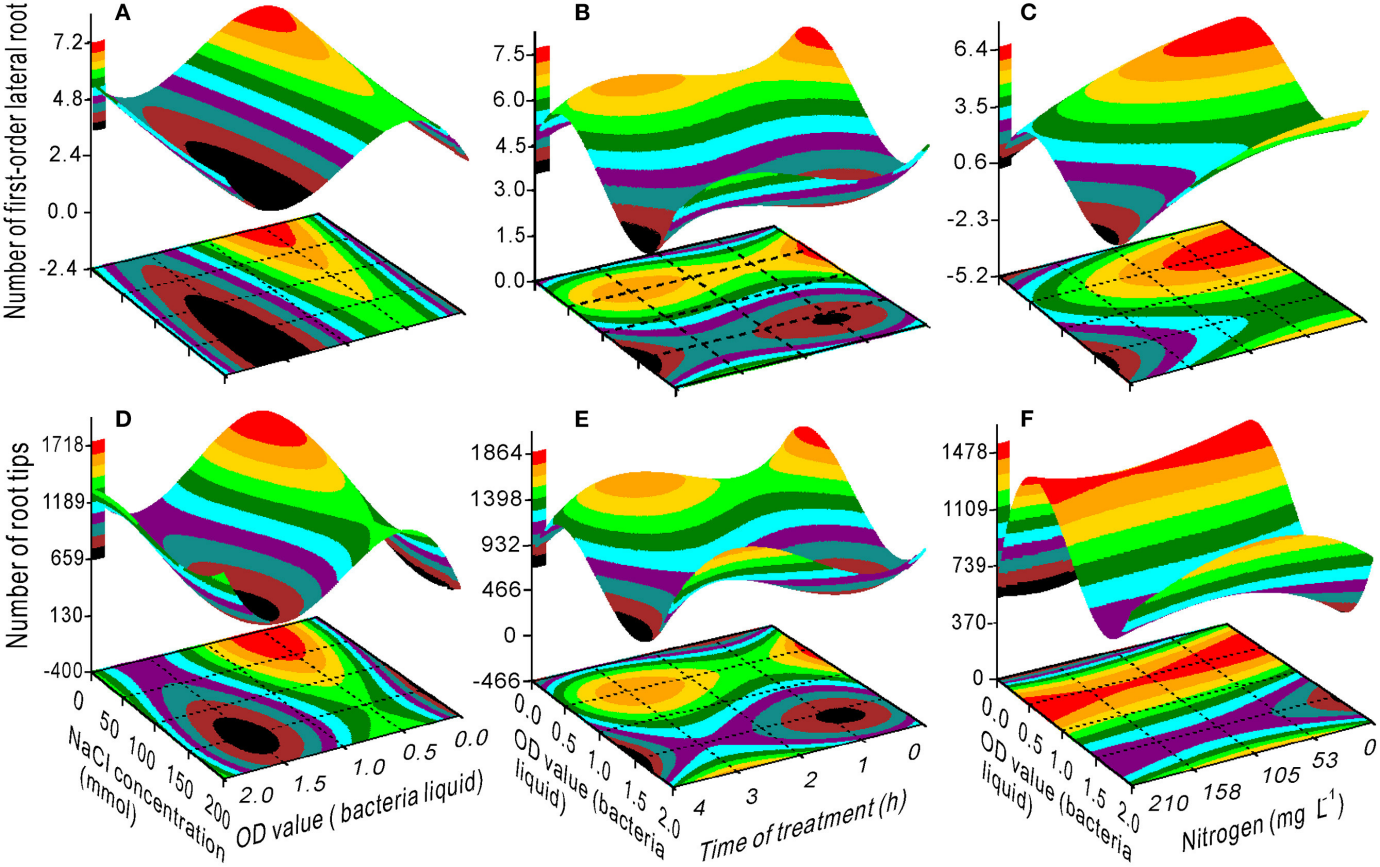
Response surface plots showing the interactions between NaCl and bacterial liquid, bacterial liquid and treatment time, and bacterial liquid and nitrogen on the number of secondary **(A–C)** and tertiary roots **(D–F)**.

The interaction effect of NaCl × bacteria on the seedling growth was evident (Figures 2, 3). The SL, TRL, RS, FW, and DW had minimum values at 200 mM NaCl and 0 OD bacterial liquid and *vice versa* due to their interaction (Figures 2A–C,E,F). The optimal bacterial liquid concentration ranged from 0.9 to 2.0 OD, which was associated with a NaCl concentration range of 60 to 125 mM for RL (Figure 2D). The 0.5 OD value of the bacterial liquid was the optimal concentration to increase the NFL and NRT when the NaCl concentration was <60 mM (Figures 3A,D) with 3 h of treatment (Figures 3B,E). However, at a 0.5 OD value, a nitrogen concentration <75 mg L^−1^ was optimal for the NFL (Figure 3C), but not significant for NRT (Figure 3F).

**Figure 4:**
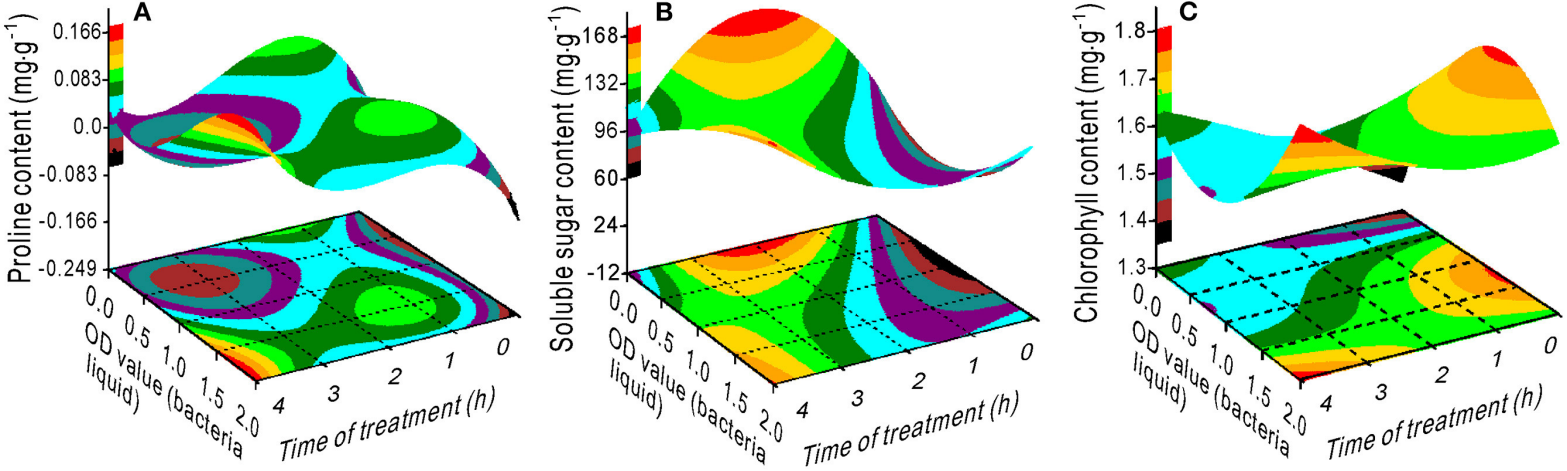
Response surface plots showing the interaction between the bacterial liquid and treatment time on the proline **(A)**, soluble sugar **(B)**, and chlorophyll **(C)** contents.

**Figure 5:**
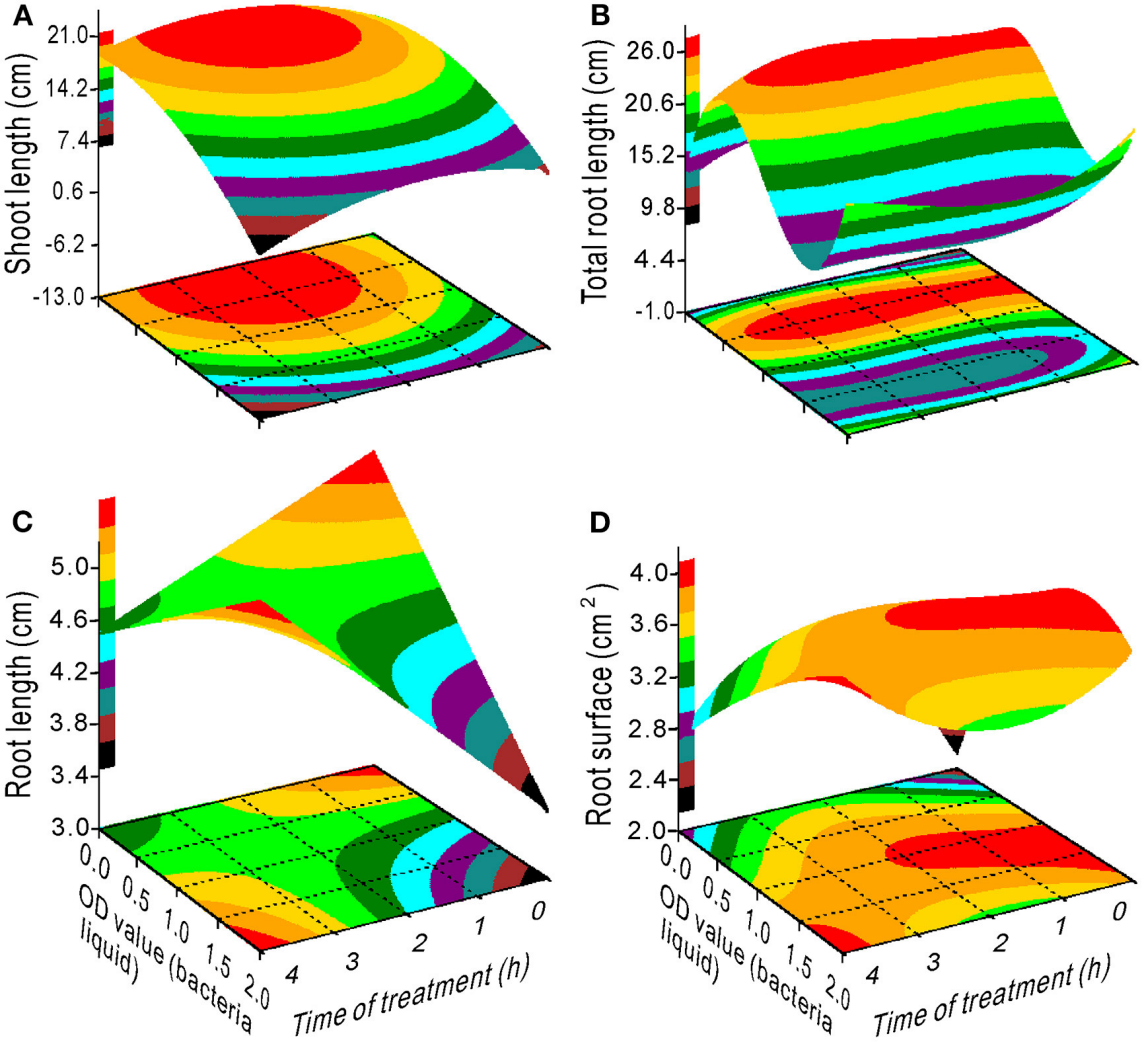
Response surface plots showing the interaction between bacterial liquid and treatment time on shoot length **(A)**, total root length **(B)**, root length **(C)**, and root surface **(D)**.

The proline content had a minimum value at an OD range of 0–1.25 and 2–4 h of treatment (Figure 4A). The SS content decreased with an increase in the OD value with a treatment time between 1 and 3 h (Figure 4B), whereas the chlorophyll content increased (Figure 4C). Maximum SL was measured at 0.5 OD with 2 h of treatment (Figure 5A) and maximum TRL at 0.5 OD (Figure 5B). The RL and RS increased with an increase in the OD when the treatment time exceeded 2.5 h (Figures 5C,D).

#### Interaction Between Nitrogen and the Other Factors

**Figure 6:**
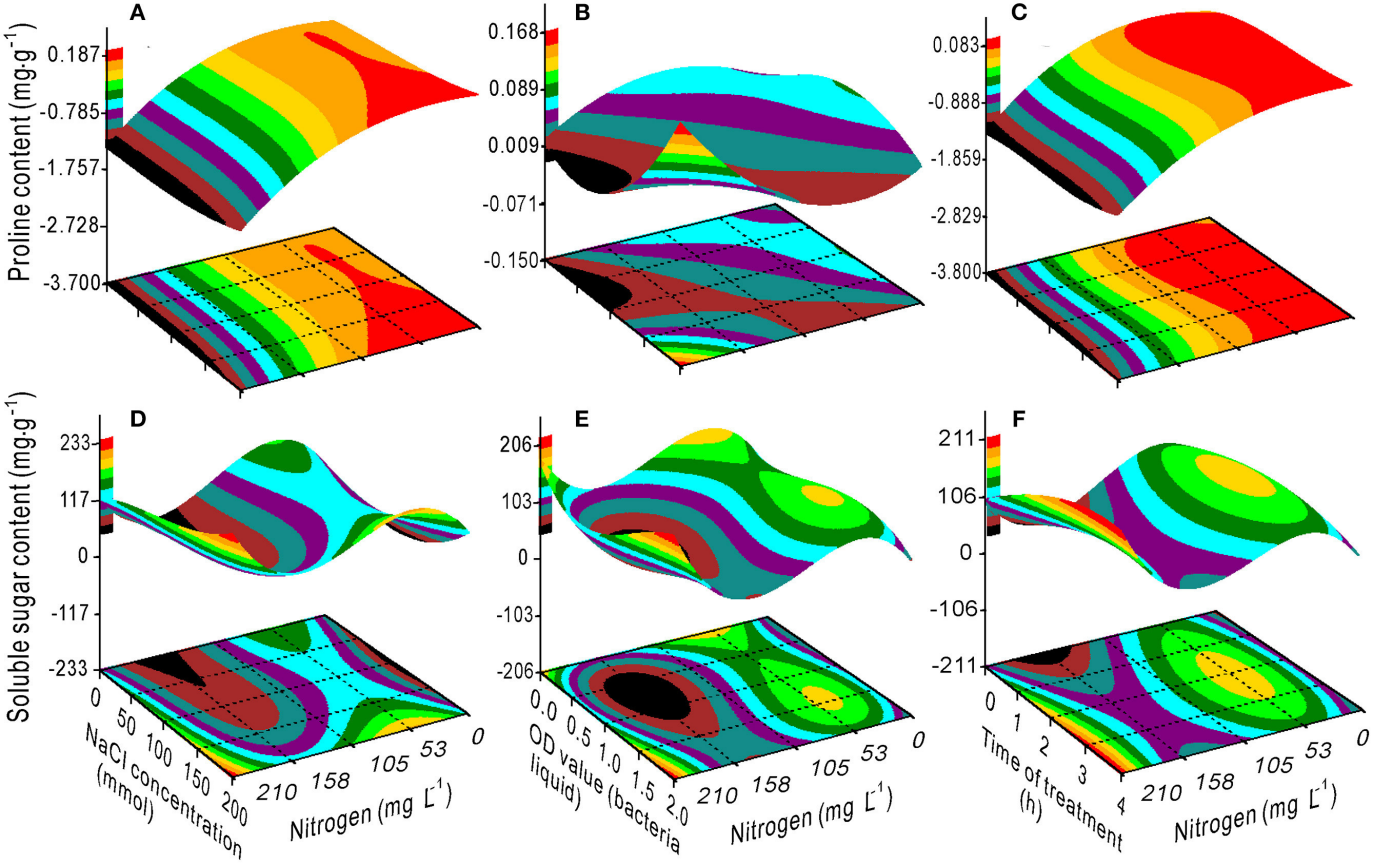
Response surface plots showing the interactions between NaCl, bacterial liquid, and treatment time with nitrogen on the proline **(A–C)** and soluble sugar **(D–F)** contents.

The variance ratio contribution of nitrogen to the proline and SS was 0.201 and 0, respectively. However, the interaction effects of NaCl × nitrogen, bacteria × nitrogen, and time × nitrogen on proline and soluble sugar were considerably greater (Table 2). The proline content decreased with an increase in nitrogen (Figures 6A,C), but increased when the OD value exceeded 1.25 (Figure 6B). The SS had a maximum value at a nitrogen concentration of 53 mg L^−1^ combined with a 1.5 OD value and a 2-h treatment time (Figures 6E,F), and a minimum value at a nitrogen concentration of ~158 mg L^−1^ combined with a 0.7 OD value (Figures 6D,E).

The SL decreased with an increase in the nitrogen concentration when the NaCl concentration < 50 mM, the OD value > 1.5 and the treatment time >3 h (Supplementary Figures 3A–C). Maximum RL was measured at a nitrogen concentration of 158 mg L^−1^ combined with an NaCl concentration < 100 mM, an OD value = 0.5, and a treatment time of 1–2 h (Supplementary Figures 3D–F). The proline and SS increased with an increase in the NaCl concentration when the treatment time was <0.5 or >3.5 h (Supplementary Figures 1A,C). The chlorophyll content increased with an increase in the NaCl concentration and treatment time (Supplementary Figure 1B). At a treatment time between 1 and 3 h, the SL, TRL, FW, and DW decreased with an increase in the NaCl concentration (Supplementary Figures 1A–C,F). The RL and RS increased with an increase in the NaCl concentration when the treatment time was >2 h (Supplementary Figures 2D,E).

**Figure 7:**
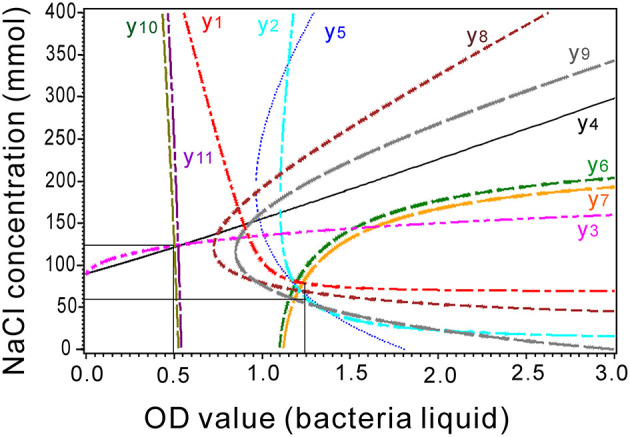
Combination of the ridge lines for the response surface models associated with the equivalent effects of NaCl concentration and bacterial liquid. y_1_, Proline content; y_2_, soluble sugar content; y_3_, chlorophyll content; y_4_, root length; y_5_, shoot length; y_6_, total root length; y_7_, root surface; y_8_, fresh weight; y_9_, dry weight; y_10_, number of first-order lateral roots; and y_11_, number of root tips of the seedlings.

The stable equilibrium point was composed of the ridge lines of the models. The intersections of the ridge lines reflect the optimal values of the independent variables for their models. The lines of the first-order lateral root (y_10_), root tips (y_11_), chlorophyll content (y_3_), and root length (y_4_) crossed at 0.5 OD bacterial liquid with 125 mM NaCl (Figure 7). The remaining lines crossed at 1.1–1.25 OD bacterial liquid with 60 mM NaCl (Figure 7).

## Discussion

The hypothesis that *Pseudomonas* sp.Y1 would significantly increase switchgrass seedling growth through an increase in the NFL and NRT was valid. A multivariate analysis of variance showed that quantifying the four factors and their interaction effects on the measured indexes was reliable.

When the NaCl concentration was 0, the SL, FW, and DW increased with an increase in the OD value (Figures 2A–F), and the TRL and RS had maximum values at a bacterial concentration of 0.5–1.25 OD (Figures 2B,E). Additionally, the bacteria had the highest variance ratio contribution to the SL, TRL, RS, NFL, and NRT (Supplementary Table 7), indicating that the bacteria promoted switchgrass cv. Blackwell growth. Furthermore, the NaCl contributed maximally to the RL and minimally to the NFL and NRT, and these results are opposite to the bacterial contributions (Supplementary Table 7). These findings demonstrate that the bacteria promoted the SL, TRL, and RS through effective proliferation of the NFL and NRT. In contrast, the TRL, RS, NFL, and NRT were reduced with an increase in the NaCl concentration (Figures 2B,E, 3A,D); however, the bacterial liquid increased these variables through an attenuation of the salinity stress in the seedlings, especially for the TRL and RS when the NaCl concentration exceeded 100 mM and the bacterial liquid concentration was higher than 1.5 OD (Figures 2B,E). A bacterial liquid concentration of 0.5 OD was optimal for root branching (NFL and NRT) (Figure 3A through Figure 3F). Additionally, the chlorophyll content increased sharply with an increase in the bacterial liquid when the NaCl concentration exceeded 150 mM (Figure 1B). This suggests that the bacteria effectively eliminated the influence of a high NaCl concentration.

Meanwhile, the optimal treatment time was 3 h in combination with the results of the variance ratio contributions (Supplementary Table 7). This may be due to switchgrass cv. Blackwell needing time to adapt to the colonization of bacteria or the long treatment time required to offset the negative effect of the salinity. The interaction effect of nitrogen and the other factors indicated that 158 mg L^−1^ nitrogen was optimal for the RL (Supplementary Figures 3D–F) and SS (Figures 6D–F).

Many studies have shown that PGPR containing ACC deaminase increases plant RL and NLR (Shahzad et al., 2010; Kim et al., 2012; Sarma and Saikia, 2014; Pan et al., 2016). We found that *Pseudomonas* sp. Y1 increases switchgrass cv. Blackwell RS and NRT, and the bacterial concentration contributed largely to the TRL, RS, NLR, and NRT (Supplementary Table 7). Bacteria containing ACC deaminase in the vicinity of roots may influence plant growth by modifying root architecture through their potential to regulate ethylene and IAA synthesis in plant roots (Shahzad et al., 2010). Ethylene and IAA play different roles in the signal that triggers lateral root initiation (LRi) (Aloni, 2013). Although ethylene is crucial for many physiological processes, this hormone inhibits root growth in numerous plant species when produced by the plants in excessive amounts (Swamy et al., 2016). Low levels of ethylene appear to enhance root initiation and growth, whereas higher levels may inhibit root elongation (Ma et al., 1998). The application of the ethylene precursor ACC at a low concentration promotes LRi (Ivanchenko et al., 2008); conversely, the application of high ethylene concentrations can inhibit LRi (Aloni, 2013). Bacteria with ACC deaminase activity metabolize ACC into α-ketobutyrate and ammonia, and therefore alleviate the toxic effect of excess ethylene on the plant (Pan et al., 2016). The ability of IAA to promote cell elongation and proliferation and positively regulate the inhibition of root cell elongation induced by ethylene is well-known (Le et al., 2001; Rahman et al., 2001). Low concentrations of IAA stimulate primary root elongation, whereas high IAA levels stimulate the formation of lateral roots, decrease primary RL, and increase root hair formation (Yang et al., 2011). However, the interaction between auxin and ethylene in root branching and root elongation is complicated (Ivanchenko et al., 2008; Wang et al., 2013). We observed that the PGPR *Pseudomonas* sp. Y1 containing ACC deaminase and IAA activity promoted switchgrass cv. Blackwell root growth, especially under high salt concentrations, which may result from the interaction between ACC deaminase and IAA.

It is commonly hypothesized that nutrient uptake is increased as a consequence of increased root surface area triggered by PGPR (Vacheron et al., 2013). A significant increase in N, P, and K uptake was observed in rice plants inoculated with PGPR under different amounts of nitrogen fertilizer. In our study, *Pseudomonas* sp. Y1 containing ACC deaminase increased the switchgrass cv. Blackwell RS and NRT. Moreover, nitrogen did not affect the TRL, RS, NLR, and NRT, but an interaction between the treatment time and bacterial liquid concentration was observed on root growth (Supplementary Table 7).

The interactions between the bacterial concentration and the treatment time, nitrogen and NaCl on the NLR and NRT, and the interaction between the bacterial concentration and treatment time on the TRL showed that a bacterial concentration of ~0.5–1.25 OD is the most effective for the NLR, NRT, and TRL. This is consistent with the findings of Kim et al. (2012), who found that *Burkholderia phytofirmans* strain PsJN at a concentration of 0.5 OD was the most effective at increasing biomass. The interaction between the bacterial concentration and NaCl on the TRL and SR indicated that the TRL and RS had maximum values at a bacterial concentration >1.0 OD. This may result from different regions of the root having varying degrees of sensitivity to the interaction between ethylene and IAA and the interaction between the different elements. Molinari et al. (2007) evaluated the stress-inducible production of proline in transgenic sugarcane (*Saccharum* sp.) and determined the osmotic adjustment, chlorophyll fluorescence, and oxidative stress.

The switchgrass cv. Blackwell endophytic *Pseudomonas* sp. Y1 promoted its maternal SL growth and increased the FW and DW. Several studies have reported that PGPR containing ACC deaminase activity improved plant growth by increasing the SL, FW, and DW (Barnawal et al., 2013, 2014). Furthermore, we discovered that the SL was positively correlated with the FW and DW (0.271, *p* < 0.0001, 0.269, *p* < 0.0001, respectively; Table 2).

Soluble sugars and proline play an important role in osmotic adjustment and may protect plants against oxidative stress (Foyer and Noctor, 2005; Molinari et al., 2007). However, the influence of PGPR on proline and SS contents is debatable. Some researchers found that the accumulation of SS and proline under stress is higher in PGPR-inoculated plants than in non-inoculated plants (Molinari et al., 2007). They explained that proline accumulation can contribute to the adjustment at the cellular level and may act as an enzyme protectant and stabilize the structure of macromolecules. In contrast, other researchers found that PGPR-inoculated plants accumulated less SS and proline than non-inoculated plants under stress (Shukla et al., 2012; Barnawal et al., 2013; Gururani et al., 2013). They hypothesized that in plants grown under salt stress, the accumulation of proline was believed to be a symptom of salt injury rather than an indication of salt tolerance (Lutts et al., 1999; Rai et al., 2003). In addition, sensitive rice cultivars under salt stress accumulated greater amounts of proline than the tolerant genotypes (Lutts et al., 1999). Our finding is consistent with the second set of observations, i.e., under salt stress, Y1 seedlings inoculated with *Pseudomonas* sp. accumulated less proline and SS than the non-inoculated ones. The interaction between nitrogen and NaCl and the treatment time indicated that the concentration of proline decreased with the addition of nitrogen, and SS had a minimum value at a nitrogen concentration of ~158 mg L^−1^. This indicates that under high nitrogen input, there is less salt toxicity, which is consistent with the findings (Guo et al., 2010; Zhou et al., 2011). In addition, Zhou et al. (2011) suggested that nitrogen input altered organic carbon allocation, with more photosynthetic products being used for growth rather than for the development of stress tolerance. Moreover, we found that SS was positively correlated with the FW and DW (0.434, *p* < 0.0001, 0.502, *p* < 0.0001, respectively; Table 2).

The chlorophyll content directly impacts the photosynthetic ability of a plant (Barkosky et al., 2000). High salinity causes a reduction in the chlorophyll content due to the suppression of specific enzymes that are responsible for the synthesis of photosynthetic pigments (Barnawal et al., 2014). Inoculation with ACC deaminase-containing bacteria has been shown to improve the pigment concentrations in plants (Barnawal et al., 2012, 2013). Furthermore, we discovered that a high bacterial concentration and a longer treatment time were more effective at increasing the chlorophyll content.

**Figure 8:**
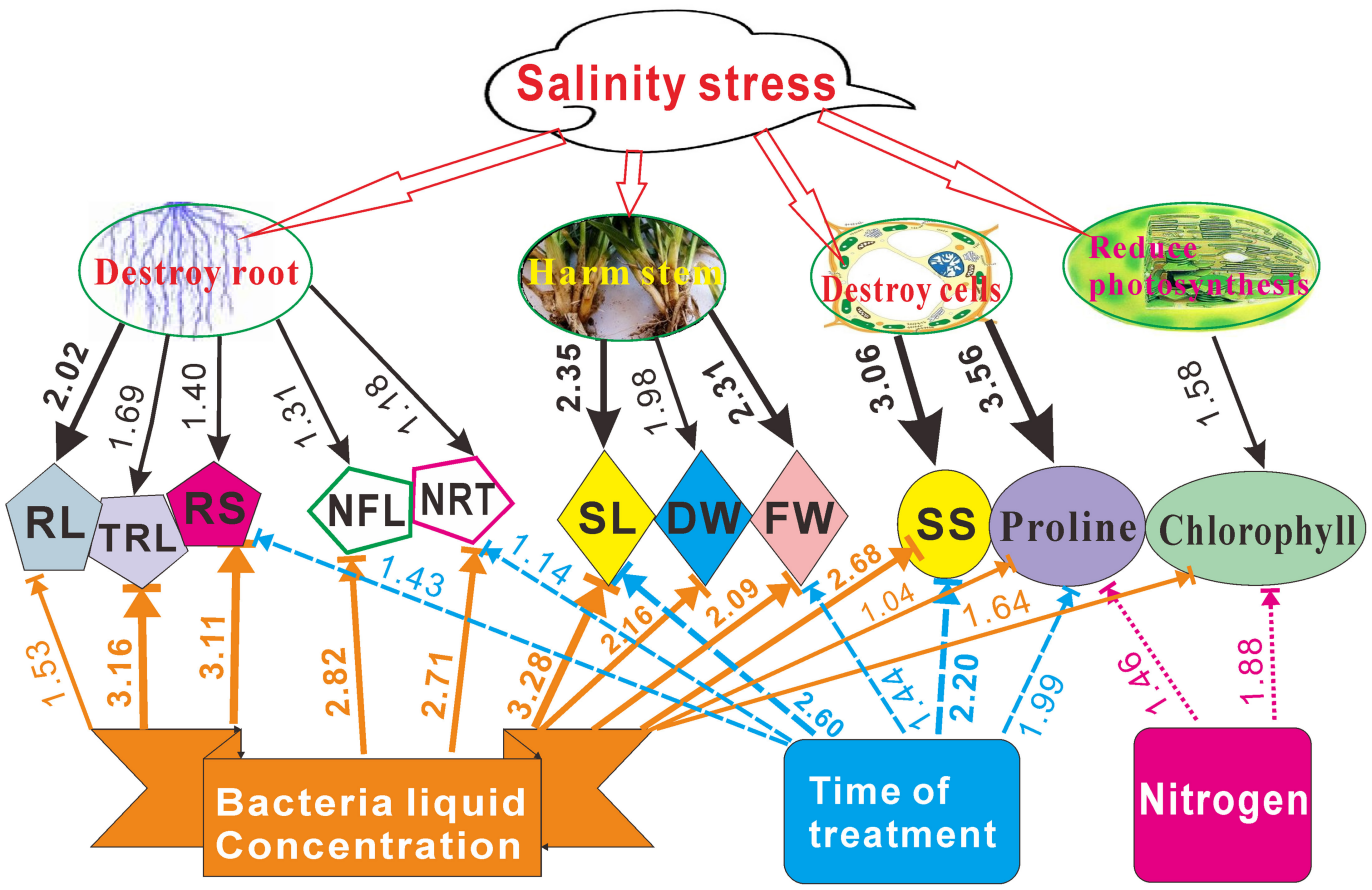
Quantitative estimation of NaCl, the bacterial liquid, treatment time and nitrogen influence on the root length (RL), total root length (TRL), root surface (RS), number of first-order lateral roots (NFL), number of root tips (NRT), shoot length (SL), dry and fresh weigh (DW and FW, respectively), soluble sugar (SS), proline and chlorophyll contents in switchgrass cv. Blackwell based on the variance ratio contributions (Supplementary Table 7). Values < 1.0 are not indicated in this chart.

Series of studies had showed that salt stress does serious harm to plant growth, such as they destroy roots and cells, reduce photosynthesis, and harm stem growth (Neves et al., 2010; Hu et al., 2013). We found that *Pseudomonas* sp. Y1 combined with appropriate nitrogen concentration can alleviate NaCl stress and promote switchgrass cv. Blackwell seedling growth. A comprehensive quantitative figure was compiled to illustrate the effect of *Pseudomonas* sp. Y1 and induced salt tolerance on the seedling growth based on the presented results (Figure 8).

## Conclusion

Rhizosphere endophytic *Pseudomonas* sp. Y1 was isolated from switchgrass cv. Blackwell under salt stress and various nitrogen concentrations. Therefore, it is likely can to be used as a plant growth promoter under stress conditions. It is effectively proliferated in the NFL and NRT in the maternal plant. Consequently, the bacteria promoted the TRL, RS, SL, FW, and DW of the plant. Additionally, the bacterial liquid increased the chlorophyll content and enhanced plant growth through an attenuation of the salinity stress. The optimal bacterial concentration, treatment time, NaCl concentration, and nitrogen concentration was 0.5–1.25 OD 600 nm, 3 h, 60–125 mM and 158 mg L^−1^, respectively. This combination was optimal to promote its maternal lateral root and seedling growth. Our findings provide an effective and sustainable approach for the development of new microbial resources and the bioprotection of plants under salt stress conditions.

## Data Availability Statement

The original contributions presented in the study are included in the article/Supplementary Material, further inquiries can be directed to the corresponding author/s.

## Author Contributions

QW designed and supervised the research project and read and approved the final manuscript. XS, NX, and TZ performed the experiments and collected the data. ZC and WZ analyzed the data and wrote the manuscript. ZG and JN revised and edited the manuscript and also provided advice on the experiment. All authors contributed to the article and approved the submitted version.

## Funding

This study was supported by National Key R&D Program of China (2017YFE0111000) and Horizon 2020 of EU-China Collaborative project (EUCLEG 727312).

## Conflict of Interest

The authors declare that the research was conducted in the absence of any commercial or financial relationships that could be construed as a potential conflict of interest.

## Publisher's Note

All claims expressed in this article are solely those of the authors and do not necessarily represent those of their affiliated organizations, or those of the publisher, the editors and the reviewers. Any product that may be evaluated in this article, or claim that may be made by its manufacturer, is not guaranteed or endorsed by the publisher.
