# Supplementary material for: Plant Growth-Promoting Rhizobacteria With ACC Deaminase Activity Enhance Maternal Lateral Root and Seedling Growth in Switchgrass
Source: Front Plant Sci. 2022 Jan 20;12:800783. doi: 10.3389/fpls.2021.800783 (PMC8811130; doi:10.3389/fpls.2021.800783)
Supplement: Supplementary file 1 [file Data_Sheet_1.docx]

**Plant growth‑promoting rhizobacteria with ACC deaminase activity enhance maternal lateral root and seedling growth in switchgrass**

**Zhao Chen^1^, Xiaomin Ma^1,2^, Wennan Zhou^1^, Jian Cui^3^ and Quanzhen Wang*^1^**

**1** Department of Grassland Science, College of Animal Sci. and Techn., Northwest A&F University, Yangling 712100, Shaanxi Province, P R China.

**2** Department of Soil Science of Temperate Ecosystems, University of Göttingen, Göttingen, Germany.

**3** Institute of Plant Science, College of life Science, Northwest A&F University, Yangling 712100, Shaanxi Province, P R China.

**To whom correspondence should be addressed**:

Dr. Quanzhen Wang

Department of Grassland Science, College of Animal Science and Technology

Northwest A & F University (<http://www.nwsuaf.edu.cn/>)

Yangling, Shanxi Province, P. R. CHINA, Phone:+86-29-87091953(o), Fax:+86-29-87092164, cellphone:+86-13759942845,

E-mail: [wangquanzhen191@163.com](mailto:wangquanzhen191@163.com)

Running head: ***Pseudomonas* sp. of switchgrass promote maternal lateral root**

**Supporting Information Captions**

**FIGURE S1| Response surface plots showing the coupling effects on contents of proline (A), chlorophyll (B) and soluble sugar (C) by NaCl concentration and time of treatment, respectively.**

**FIGURE S2| Response surface plots showing the coupling effects on shoot length (A), total root length (B), fresh weight (C), root length (D), root surface (E) and dry weight (F) by NaCl concentration and time of treatment, respectively.**

**FIGURE S3| Response surface plots showing the coupling effects on shoot length (A, B and C) and root length (D, E and F) respectively due to NaCl, bacteria liquid and time of treatment with nitrogen.**

**TABLE S1| Coefficients of the models concerning to dependent variables with NaCl concentration (X_1_) and bacteria liquid concentration (X_2_).**

**TABLE S2| Coefficients of the models concerning to dependent variables with NaCl concentration (X_1_) and treatment time (X_3_).**

**TABLE S3| Coefficients of the models concerning to dependent variables with bacteria liquid concentration (X_2_) and treatment time (X_3_).**

**TABLE S4| Coefficients of the models concerning to dependent variables with NaCl concentration (X_1_) and nitrogen content (X_4_).**

**TABLE S5| Coefficients of the models concerning to dependent variables with bacteria liquid concentration (X_2_) and nitrogen content (X_4_).**

**TABLE S6|Coefficients of the models concerning to dependent variables with treatment time (X_3_) and nitrogen content (X_4_).**

**TABLE S7| The variance ratio contributions to proline content, contents of soluble sugar and chlorophyll content, shoot lengths, root length, total root length, root surface and fresh weight, dry weight, water content, number of first-order lateral root and root tips of the seedlings due to NaCl, bacteria concentration, time of treatment with bacteria, nitrogen concentration and their cross products.**


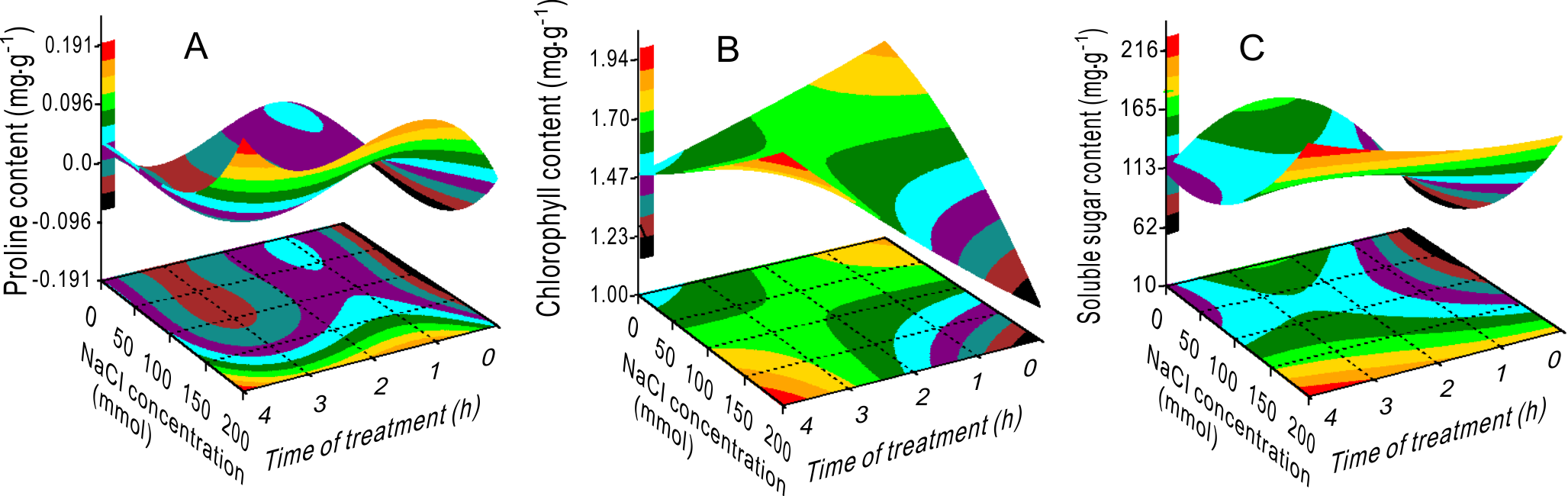


**FIGURE S1| Response surface plots showing the coupling effects on contents of proline (A), chlorophyll (B) and soluble sugar (C) by NaCl concentration and time of treatment, respectively.**


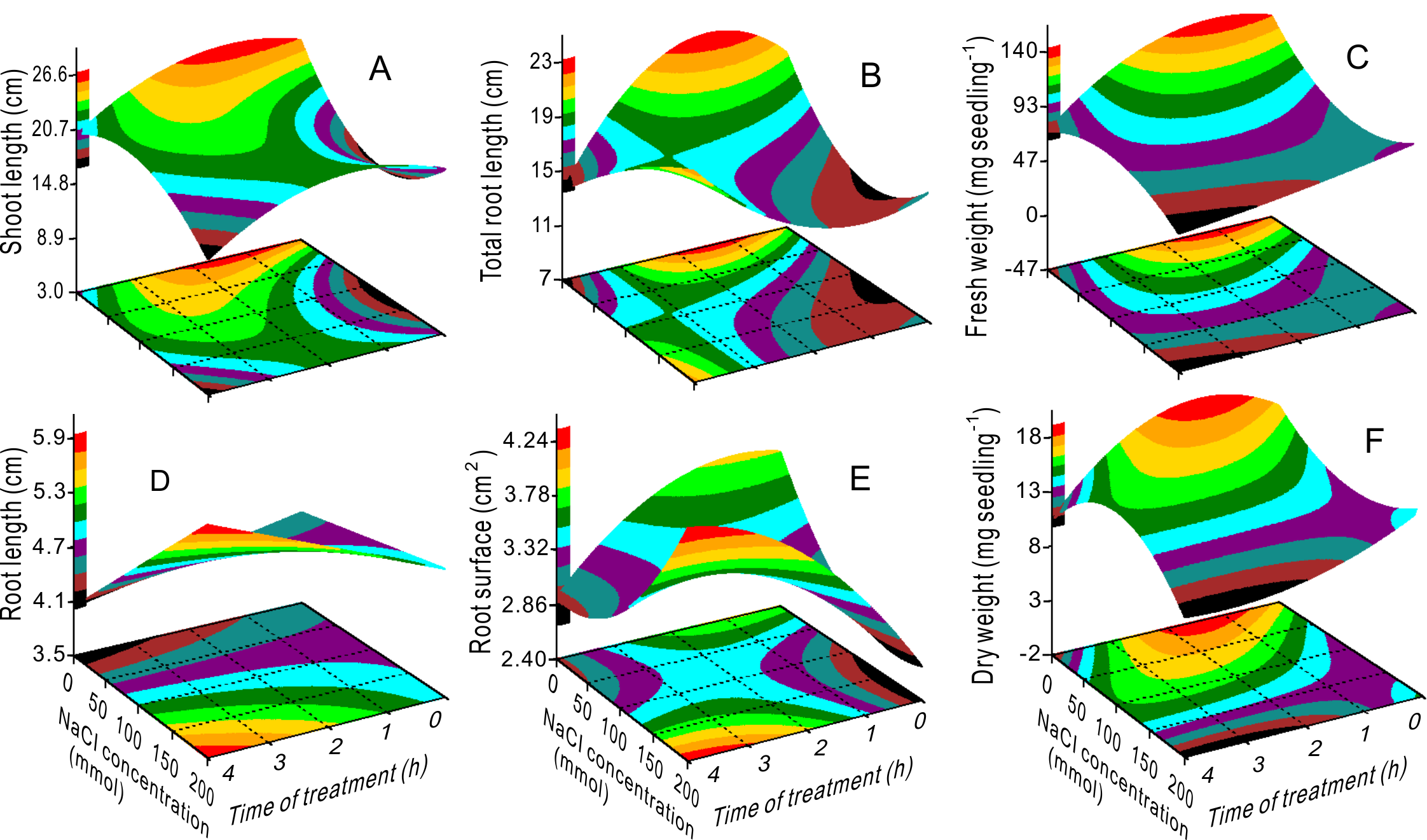


**FIGURE S2| Response surface plots showing the coupling effects on shoot length (A), total root length (B), fresh weight (C), root length (D), root surface (E) and dry weight (F) by NaCl concentration and time of treatment, respectively.**


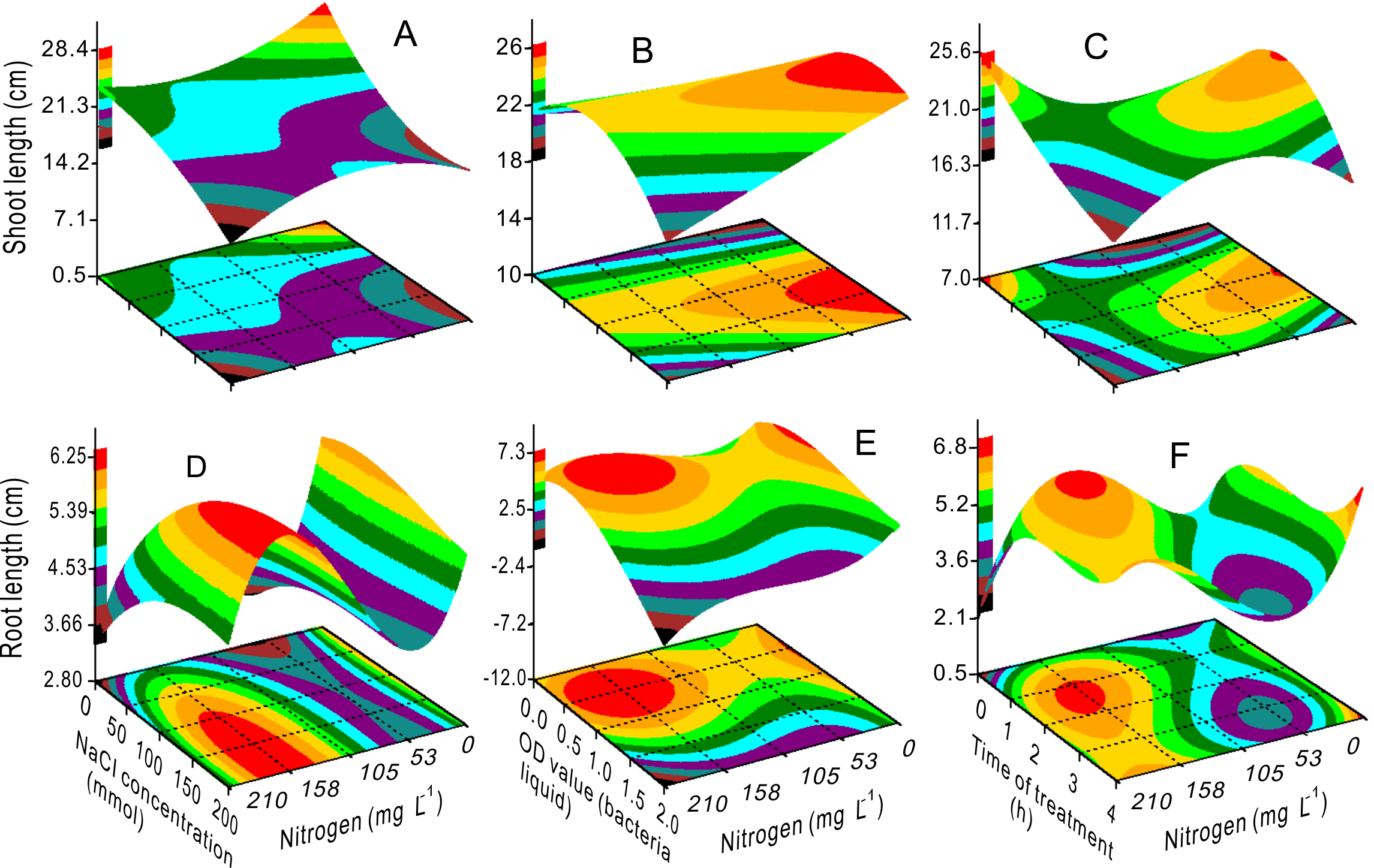


**FIGURE S3| Response surface plots showing the coupling effects on shoot length (A, B and C) and root length (D, E and F) respectively due to NaCl, bacteria liquid and time of treatment with nitrogen.**

**TABLE S1| Coefficients of the models concerning to dependent variables with NaCl concentration (X_1_) and bacteria liquid concentration (X_2_).**

| Parameter | Intercept | *X*_1_ | *X*_2_ | *X*_1_·*X*_2_ | *X*_1_·*X*_1_ | *X*_2_·*X*_2_ | *X*_1_·*X*_1_·*X*_2_ | *X*_1_·*X*_2_·*X*_2_ | *X*_1_·*X*_1_·*X*_1_ | *X*_2_·*X*_2_·*X*_2_ | *Pr > F* |
| --- | --- | --- | --- | --- | --- | --- | --- | --- | --- | --- | --- |
| *Y*_1_ | 0.026404 | 0.000495 | -0.15266 | -8.82E-06 | -0.00091 | 0.26952 | 9.09E-07 | 0.000418 | 5.41E-08 | -0.09856 | <0.0001 |
| *Y*_2_ | 129.881 | -0.42963 | -46.88517 | 0.0033413 | 0.006133 | 19.58389 |  |  |  |  | 0.0059 |
| *Y*_3_ | 1.506421 | 0.003676 | 0.3202496 | -2.68E-05 | -0.00526 | -0.08881 | 2.81E-05 | 0.000424 |  |  | 0.0060 |
| *Y*_4_ | 4.0438 | 0.0297 | -0.1581 | -0.0001 | -0.0141 | 0.1469 | 0.0001 | -0.0047 |  |  | 0.0041 |
| *Y*_5_ | 20.8434 | -0.02084 | 8.7634099 | 3.538E-05 | -0.01961 | -2.44476 | 0.000112 | -0.01167 |  |  | <0.0001 |
| *Y*_6_ | 16.72826 | -0.04024 | 16.301729 | 9.193E-05 | -0.06263 | -7.45397 | 8.91E-05 | 0.031044 |  |  | 0.0001 |
| *Y*_7_ | 2.901049 | -0.01223 | 6.6453585 | 0.0001205 | -0.00748 | -7.72903 | 1.18E-05 | 0.003665 | -3.5E-07 | 2.285062 | <0.0001 |
| *Y*_8_ | 0.086644 | 0.000285 | 0.0389629 | -2.44E-06 | -0.00049 | 0.002962 | 3.26E-06 | -0.00016 |  |  | <0.0001 |
| *Y*_9_ | 0.01233 | 6.33E-05 | 0.0075387 | -3.57E-07 | -7.4E-05 | -0.00129 | 3.79E-07 | -1.1E-05 |  |  | 0.0097 |
| *Y*_10_ | 4.739642 | -0.00572 | 10.313094 | -5.05E-06 | -0.00667 | -12.9842 | -6E-08 | 0.004004 | 8E-08 | 4.01052 | 0.0003 |
| *Y*_11_ | 963.9668 | 2.09943 | 3067.8545 | -0.065025 | -2.03668 | -3736.73 | 0.000527 | 1.302946 | 0.000247 | 1144.319 | 0.0027 |

Note y_1_: proline content, y_2_: soluble sugar content, y_3_: chlorophyll content, y_4_: root length, y_5_: shoot length, y_6_: total root length, y_7_: root surface, y_8_: fresh weight, y_9_: dry weight, y_10_: number of first-order lateral roots and y_11_: number of root tips of the seedlings.

**TABLE S2| Coefficients of the models concerning to dependent variables with NaCl concentration (X_1_) and treatment time (X_3_).**

| Parameter | Intercept | *X*_1_ | *X*_3_ | *X*_1_·*X*_3_ | *X*_1_·*X*_1_ | *X*_3_·*X*_3_ | *X*_1_·*X*_1_·*X*_3_ | *X*_1_·*X*_3_·*X*_3_ | *X*_1_·*X*_1_·*X*_1_ | *X*_3_·*X*_3_·*X*_3_ | *Pr > F* |
| --- | --- | --- | --- | --- | --- | --- | --- | --- | --- | --- | --- |
| *Y*_1_ | -0.06326 | 0.000325 | 0.1935413 | -8.8E-06 | -7.7E-06 | -0.11429 | 3.74E-07 | -1.9E-06 | 5.41E-08 | 0.018067 | <0.0001 |
| *Y*_2_ | 51.477 | -0.18037 | 84.866761 | 0.0040811 | -0.41488 | -17.5159 | -0.00039 | 0.100421 |  |  | 0.0030 |
| *Y*_3_ | 1.807777 | -0.00142 | -0.094588 | -9.34E-06 | 0.000742 | 0.00314 | 2.52E-06 | 1.86E-05 |  |  | 0.0053 |
| *Y*_5_ | 25.44728 | -0.14592 | 1.8436568 | 0.0005777 | 0.051944 | -0.7961 | -0.00024 | -0.00045 |  |  | <0.0001 |
| *Y*_6_ | 20.39638 | -0.10075 | 3.8010819 | 0.0003862 | -0.00395 | -1.3773 | -0.00012 | 0.010841 |  |  | 0.0028 |
| *Y*_7_ | 3.759753 | -0.01341 | 0.126382 | 3.673E-05 | 0.003057 | -0.084 |  |  |  |  | 0.0039 |
| *Y*_8_ | 0.13319 | -0.00079 | 0.0147268 | 2.504E-06 | 0.000137 | -0.00748 | -1.3E-06 | 4.27E-05 |  |  | 0.0005 |

Note y_1_: proline content, y_2_: soluble sugar content, y_3_: chlorophyll content, y_4_: root length, y_5_: shoot length, y_6_: total root length, y_7_: root surface and y_8_: fresh weight of the seedlings.

**TABLE S3| Coefficients of the models concerning to dependent variables with bacteria liquid concentration (X_2_) and treatment time (X_3_).**

| Parameter | Intercept | *X*_2_ | *X*_3_ | *X*_2_·*X*_3_ | *X*_2_·*X*_2_ | *X*_3_·*X*_3_ | *X*_2_·*X*_2_·*X*_3_ | *X*_2_·*X*_3_·*X*_3_ | *X*_2_·*X*_2_·*X*_2_ | *X*_3_·*X*_3_·*X*_3_ | *Pr > F* |
| --- | --- | --- | --- | --- | --- | --- | --- | --- | --- | --- | --- |
| *Y*_1_ | -0.01504 | -0.20762 | 0.2087314 | 0.2872786 | -0.03103 | -0.12076 | 0.010574 | 0.008004 | -0.09856 | 0.018067 | 0.0008 |
| *Y*_2_ | 90.25 | -74.9872 | 77.633942 | 44.174916 | -23.6322 | -19.2939 | -13.1152 | 13.73448 |  |  | <0.0001 |
| *Y*_3_ | 1.365648 | 0.647433 | 0.0809192 | -0.249744 | -0.26702 | -0.00323 | 0.106173 | 0.01021 |  |  | 0.0973 |
| *Y*_4_ | 5.4044 | -0.9924 | -0.2504 | 0.3744 |  |  |  |  |  |  | 0.0067 |
| *Y*_5_ | 16.51701 | 9.479606 | 3.9162542 | -3.495035 | -0.5719 | -0.83702 |  |  |  |  | <0.0001 |
| *Y*_6_ | 8.017192 | 63.64344 | 3.2799628 | -69.02529 | -7.83556 | 0.505275 | 1.564464 | 0.999525 | 20.39825 | -0.26323 | <0.0001 |
| *Y*_7_ | 2.123522 | 2.488129 | 1.0323072 | -0.85033 | -1.08409 | -0.21862 | 0.177551 | 0.170951 |  |  | 0.0002 |
| *Y*_10_ | 4.921695 | 10.82559 | -1.961468 | -13.24516 | -0.44474 | 1.260871 | 0.331343 | -0.05272 | 4.01052 | -0.19849 | 0.0022 |
| *Y*_11_ | 1107.593 | 3124.725 | -826.6717 | -3805.876 | -35.8407 | 532.0122 | 99.42043 | -31.3103 | 1144.319 | -85.9375 | 0.0100 |

Note y_1_: proline content, y_2_: soluble sugar content, y_3_: chlorophyll content, y_4_: root length, y_5_: shoot length, y_6_: total root length, y_7_: root surface, y_8_: fresh weight, y_9_: dry weight, y_10_: number of first-order lateral roots and y_11_: number of root tips of the seedlings.

**TABLE S4| Coefficients of the models concerning to dependent variables with NaCl concentration (X_1_) and nitrogen content (X_4_).**

| Parameter | Intercept | *X*_1_ | *X*_4_ | *X*_1_·*X*_4_ | *X*_1_·*X*_1_ | *X*_4_·*X*_4_ | *X*_1_·*X*_1_·*X*_4_ | *X*_1_·*X*_4_·*X*_4_ | *X*_1_·*X*_1_·*X*_1_ | *X*_4_·*X*_4_·*X*_4_ | *Pr > F* |
| --- | --- | --- | --- | --- | --- | --- | --- | --- | --- | --- | --- |
| *Y*_1_ | -0.02453 | 0.00077 | 0.0024078 | -1.04E-05 | -8.6E-06 | -2.6E-05 | 2.37E-08 | 2.42E-08 | 5.41E-08 | 7.57E-08 | <0.0001 |
| *Y*_2_ | 89.39321 | -0.65539 | 3.2822204 | 0.0001002 | 0.004402 | -0.04432 | -2.6E-05 | 1.15E-05 | 2.1E-05 | 0.000135 | <0.0001 |
| *Y*_3_ | 1.796216 | -0.00109 | -0.009456 | 4.543E-05 | -4.3E-05 | 0.00011 | 1.92E-07 | 6.6E-08 | -2.5E-07 | -3.1E-07 | 0.0548 |
| *Y*_4_ | 5.814202 | 0.00499 | -0.081551 | -6.47E-05 | 0.000152 | 0.000901 | -1.6E-07 | -3.1E-07 | 1.35E-07 | -2.7E-06 | 0.0190 |
| *Y*_5_ | 28.40176 | -0.12683 | -0.08308 | 0.0003759 | 0.001304 | 0.000288 | -2.7E-06 | -3.3E-06 |  |  | 0.0001 |
| *Y*_8_ | 0.20622 | -0.00081 | -0.002939 | -2.07E-06 | 1.45E-05 | 2.45E-05 | -8.5E-09 | -4.7E-08 | 1.11E-08 | -6.1E-08 | <0.0001 |
| *Y*_9_ | 0.026315 | -9.2E-05 | -0.000354 | -2.73E-08 | 1.85E-06 | 3.15E-06 | -1E-09 | -6E-09 | 2E-10 | -8.2E-09 | 0.0144 |

Note y_1_: proline content, y_2_: soluble sugar content, y_3_: chlorophyll content, y_4_: root length, y_5_: shoot length, y_6_: total root length, y_7_: root surface, y_8_: fresh weight and y_9_: dry weight of the seedlings.

**TABLE S5| Coefficients of the models concerning to dependent variables with bacteria liquid concentration (X_2_) and nitrogen content (X_4_).**

| Parameter | Intercept | *X*_2_ | *X*_4_ | *X*_2_·*X*_4_ | *X*_2_·*X*_2_ | *X*_4_·*X*_4_ | *X*_2_·*X*_2_·*X*_4_ | *X*_2_·*X*_4_·*X*_4_ | *X*_2_·*X*_2_·*X*_2_ | *X*_4_·*X*_4_·*X*_4_ | *Pr > F* |
| --- | --- | --- | --- | --- | --- | --- | --- | --- | --- | --- | --- |
| *Y*_1_ | 0.027625 | 0.10566 | 0.0009959 | -0.050819 | -0.00221 | -4.7E-06 | 0.000642 | 5.98E-06 |  |  | <0.0001 |
| *Y*_2_ | 69.82559 | -68.438 | 3.9129021 | 141.24401 | -1.37563 | -0.04552 | 0.419209 | 0.002846 | -53.6425 | 0.000135 | <0.0001 |
| *Y*_3_ | 1.59932 | 0.164472 | -0.003465 | -0.050669 | 0.000384 | 1.59E-05 |  |  |  |  | 0.0153 |
| *Y*_4_ | 5.797631 | 3.99502 | -0.072426 | -4.676495 | -0.01134 | 0.000885 | 0.009171 | -1.5E-05 | 1.172099 | -2.7E-06 | 0.0277 |
| *Y*_5_ | 17.89264 | 10.43627 | 0.0176887 | -3.495035 | -0.02132 | -1.1E-05 |  |  |  |  | <0.0001 |
| *Y*_6_ | 11.03539 | 47.78217 | 0.1536025 | -63.19299 | 0.063737 | -0.00233 | -0.03046 | 2.86E-05 | 20.39825 | 7.64E-06 | <0.0001 |
| *Y*_7_ | 2.265906 | 5.93046 | 0.0290597 | -7.305103 | -0.00103 | -0.00038 | -0.00099 | 1.99E-05 | 2.285062 | 1.19E-06 | <0.0001 |
| *Y*_8_ | 0.091782 | -0.02261 | -0.000134 | 0.0259804 | 0.000505 | 1.74E-07 | -0.0004 | 7.39E-07 |  |  | 0.0556 |
| *Y*_10_ | 4.787704 | 8.006617 | -0.010529 | -11.94791 | 0.021736 | 4.39E-05 | -0.0071 | -2.5E-05 | 4.01052 | -4E-08 | 0.0010 |
| *Y*_11_ | 1072.704 | 2188.24 | -4.723905 | -3389.303 | 10.32278 | 0.002169 | -2.41872 | -0.01925 | 1144.319 | 0.00006 | 0.0041 |

Note y_1_: proline content, y_2_: soluble sugar content, y_3_: chlorophyll content, y_4_: root length, y_5_: shoot length, y_6_: total root length, y_7_: root surface, y_8_: fresh weight, y_9_: dry weight, y_10_: number of first-order lateral roots and y_11_: number of root tips of the seedlings.

**TABLE S6|Coefficients of the models concerning to dependent variables with treatment time (X_3_) and nitrogen content (X_4_).**

| Parameter | Intercept | *X*_3_ | *X*_4_ | *X*_3_·*X*_4_ | *X*_3_·*X*_3_ | *X*_4_·*X*_4_ | *X*_3_·*X*_3_·*X*_4_ | *X*_3_·*X*_4_·*X*_4_ | *X*_3_·*X*_3_·*X*_3_ | *X*_4_·*X*_4_·*X*_4_ | *Pr > F* |
| --- | --- | --- | --- | --- | --- | --- | --- | --- | --- | --- | --- |
| *Y*_1_ | -0.14475 | 0.283124 | 0.0038022 | -0.131168 | -0.0015 | -3.1E-05 | 0.000176 | 3.92E-06 | 0.018067 | 7.57E-08 | <0.0001 |
| *Y*_2_ | 49.89291 | 30.7302 | 3.5723017 | -4.158164 | -0.12178 | -0.04531 | 0.002261 | 0.001085 | -0.70468 | 0.000135 | 0.0003 |
| *Y*_4_ | 4.232187 | 2.391216 | -0.048989 | -1.352747 | -0.00578 | 0.000748 | -0.00188 | 6.7E-05 | 0.22869 | -2.7E-06 | 0.0244 |
| *Y*_5_ | 17.72942 | 7.057205 | -0.027386 | -1.739094 | -0.01177 | 0.000308 | 0.00948 | -0.00017 |  |  | 0.0101 |
| *Y*_6_ | 4.559788 | 15.72803 | 0.1293885 | -3.02071 | -0.15935 | -4.7E-05 | 0.027524 | 9.47E-05 |  |  | 0.0043 |
| *Y*_7_ | 1.483106 | 2.210405 | 0.020466 | -0.430871 | -0.02251 | -2.9E-05 | 0.003645 | 2.3E-05 |  |  | 0.0013 |

Note y_1_: proline content, y_2_: soluble sugar content, y_3_: chlorophyll content, y_4_: root length, y_5_: shoot length, y_6_: total root length and y_7_: root surface of the seedlings.

**TABLE S7| The variance ratio contributions to proline content, contents of soluble sugar and chlorophyll content, shoot lengths, root length, total root length, root surface and fresh weight, dry weight, water content, number of first-order lateral root and root tips of the seedlings due to NaCl, bacteria concentration, time of treatment with bacteria, nitrogen concentration and their cross products.**

|  | Proline | Soluble sugar | Chlorophyll | Shoot lengths | Root length | Total root length | Root surface | Fresh weight | Dry weight | First-order lateral root | Root tips | Total |
| --- | --- | --- | --- | --- | --- | --- | --- | --- | --- | --- | --- | --- |
| NaCl | 2.951 | 2.602 | 1.057 | 1.731 | 1.700 | 1.222 | 0.982 | 1.436 | 1.184 | 0.877 | 0.851 | **16.593** |
| Bacteria | 0.143 | 1.803 | 1.308 | 2.705 | 1.017 | 2.717 | 2.693 | 1.428 | 1.797 | 2.516 | 2.356 | **20.483** |
| Time of treatment | 1.239 | 1.435 | 0.448 | 1.911 | 0 | 0 | 0.699 | 1.010 | 0.238 | 0.248 | 0.675 | **7.903** |
| Nitrogen | 0.201 | 0 | 1.729 | 0 | 0 | 0 | 0 | 0.103 | 0 | 0 | 0 | **2.033** |
| NaCl×bacteria | 1.233 | 0.512 | 1.262 | 0.901 | 0.886 | 0.971 | 0.921 | 2.095 | 1.592 | 0.187 | 0 | **10.560** |
| NaCl×time | 0.415 | 0.733 | 0.945 | 1.376 | 0.310 | 1.457 | 1.592 | 1.356 | 1.456 | 1.943 | 1.489 | **13.072** |
| Bacteria×time | 1.337 | 2.601 | 0.739 | 0.543 | 0.891 | 1.118 | 1.218 | 0.561 | 0.270 | 0.689 | 0.371 | **10.338** |
| NaCl×nitrogen | 1.997 | 1.480 | 0.929 | 1.456 | 0.749 | 0.387 | 0 | 1.774 | 1.721 | 0.495 | 0.468 | **11.456** |
| Bacteria×nitrogen | 2.806 | 2.169 | 0 | 2.035 | 1.312 | 0.563 | 0.355 | 1.294 | 0.342 | 0.934 | 1.740 | **13.550** |
| Time×nitrogen | 2.745 | 1.276 | 0 | 2.188 | 1.267 | 1.168 | 1.588 | 0.684 | 0 | 1.723 | 0.924 | **13.563** |
| Total | **15.067** | **14.611** | **8.417** | **14.846** | **8.132** | **9.603** | **10.048** | **11.741** | **8.6** | **9.612** | **8.874** |  |
